# Microevolution of *Helicobacter pylori* during Prolonged Infection of Single Hosts and within Families

**DOI:** 10.1371/journal.pgen.1001036

**Published:** 2010-07-22

**Authors:** Giovanna Morelli, Xavier Didelot, Barica Kusecek, Sandra Schwarz, Christelle Bahlawane, Daniel Falush, Sebastian Suerbaum, Mark Achtman

**Affiliations:** 1 Department of Molecular Biology, Max-Planck Institute for Infection Biology, Berlin, Germany; 2 Department of Statistics, University of Warwick, Warwick, United Kingdom; 3 Institute of Medical Microbiology and Hospital Epidemiology, Hannover Medical School, Hannover, Germany; 4 Environmental Research Institute and Department of Microbiology, University College Cork, Cork, Ireland; Fred Hutchinson Cancer Research Center, United States of America

## Abstract

Our understanding of basic evolutionary processes in bacteria is still very limited. For example, multiple recent dating estimates are based on a universal inter-species molecular clock rate, but that rate was calibrated using estimates of geological dates that are no longer accepted. We therefore estimated the short-term rates of mutation and recombination in *Helicobacter pylori* by sequencing an average of 39,300 bp in 78 gene fragments from 97 isolates. These isolates included 34 pairs of sequential samples, which were sampled at intervals of 0.25 to 10.2 years. They also included single isolates from 29 individuals (average age: 45 years) from 10 families. The accumulation of sequence diversity increased with time of separation in a clock-like manner in the sequential isolates. We used Approximate Bayesian Computation to estimate the rates of mutation, recombination, mean length of recombination tracts, and average diversity in those tracts. The estimates indicate that the short-term mutation rate is 1.4×10^−6^ (serial isolates) to 4.5×10^−6^ (family isolates) per nucleotide per year and that three times as many substitutions are introduced by recombination as by mutation. The long-term mutation rate over millennia is 5–17-fold lower, partly due to the removal of non-synonymous mutations due to purifying selection. Comparisons with the recent literature show that short-term mutation rates vary dramatically in different bacterial species and can span a range of several orders of magnitude.

## Author Summary

Mutation rates in bacteria have generally been considered to be much slower than in viruses. This is partly because estimates of long-term mutation rates for the evolution of distinct species have been inappropriately used for dating divergence within species. Furthermore, the most commonly used long-term mutation rate is based on geological dates that are no longer accepted. In addition, only few short-term mutation rates have been calculated within bacterial species, and these differ with the species by several orders of magnitude. Here, we provide robust estimates for short-term mutation and recombination rates within *Helicobacter pylori*, a bacterium that commonly infects the human gastric mucosa, based on serial isolates from long-term infections and on differences between isolates from multiple family members. These short-term mutation rates are 5–17-fold faster than long-term mutation rates in *H. pylori* that have been calibrated by parallel ancient migrations of humans. Short-term mutation rates in bacteria, including those for *H. pylori*, can be quite fast, partially overlapping with those for viruses. Future calculations of ages of bacterial species will need to account for dramatic differences in mutation rate between species and for dramatic differences between short- and long-term mutation rates.

## Introduction

When did modern pathogenic bacteria evolve? Current wisdom teaches that 10,000–50,000 years have elapsed since a variety of genetically highly monomorphic bacterial pathogens evolved from their last common ancestors [1]–[6] and the ages of pathogenic bacteria with greater levels of genetic diversity have been estimated as reflecting millions of years of evolution [7], [8]. Age estimates for bacteria are higher than those of viruses, many of which appeared a few hundred years ago [9], primarily because many bacterial estimates are based on a supposedly universal molecular clock rate, μ_S_, for synonymous polymorphisms in genes that encode proteins. In 1987, Ochman and Wilson calibrated this clock rate as 3.4×10^−9^ per nucleotide per year by dating the split between *Escherichia coli* and *Salmonella enterica* within the framework of a universal clock rate for bacterial rRNA sequences [10]. The divergence between *E. coli* and *S. enterica* was equated with the age of mammals, estimated as ∼160 Myr. However, the validity of this molecular clock rate for dating bacterial evolution is highly questionable.

**Figure 1:**
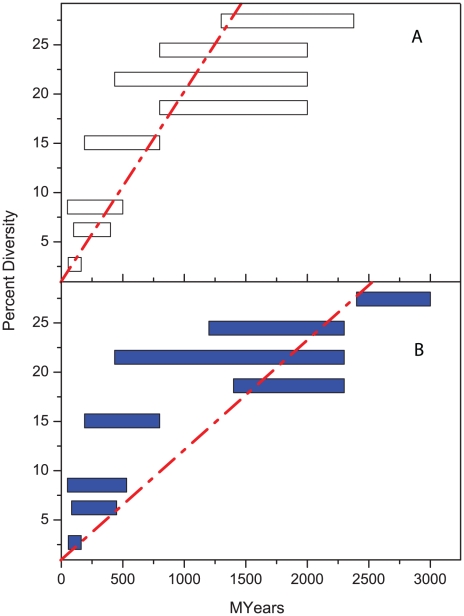
Percentage diversity in rRNA versus age (million years). (A) original correlation by Ochman and Wilson [10]. (B) lack of good correlation according to modern estimates of age ranges (Table 1).

Some of the geological dates used to calibrate the rRNA clock rate have since been revised (Table 1). These revisions are so drastic that the original linear regression of diversity with time [10] is no longer valid [11] (Figure 1). Furthermore, the estimate of ∼160 Mya for the age of the split between *E. coli* and *S. enterica* depends on the assumption that *E. coli* is specific for mammalian hosts, unlike *S. enterica* which infects reptiles as well as mammals. But *E. coli* can be readily isolated from reptiles or birds [12], which invalidates this argument. An independent recent study also dates the split between *E. coli* and *S. enterica* at 57–176 Mya on the basis of long-term phylogenies of protein-encoding sequences [13]. However, both this recent estimate and the original estimate of Ochman and Wilson share the problem that geological events that occurred billions of years ago are extrapolated to speciation events that supposedly occurred ∼100 Mya, which implicitly assumes that molecular clock rates are linear over large time scales for diverse microorganisms. This is unlikely to be the case (see below). The use of such long-term clock rates is even more problematical for age estimates of divergence within genetically monomorphic or recently emerged pathogens [1]–[6], which require extrapolations over a further three to four orders of magnitude.

**Table 1:** Comparisons of dating used by Ochman and Wilson, 1987 [10] with current estimates.

Time Point	Event	Old Estimate (MYa)	*Current Estimate (MYa)*	*Citation*
A	*Cyanobacteria*	>1,300	>2,400	[64]
B-C	Photosynthetic eukaryotes	>800	>1,200	[65]
C	Oxygen	<2,000	2,300	[66]
D	Oxidative eukaryotes	>800	>1,400	[67]
E	High concentration O_2_	<800		
F	Light organs	>50		
G	Eyes	<500	>531	[68]
H	Land plants	<400	450	
I	Mammals	<150	>162	[69]
J	Legumes	>100	>84	[70]

Long-term clock rates are now thought to accelerate by one to two orders of magnitude for recent events [14], [15]. Furthermore, clock rates for genetic diversity between species should not be used for dating within a species. Diversity between species represents fixation events whereas diversity within a species reflects the accumulation of polymorphisms [16], [17]. Finally, molecular clock rates probably vary between different bacterial species, which can differ by up to two orders of magnitude in their relative ratios of divergence of rRNA to protein-encoding genes [18]. As a result of these considerations, almost all age estimates for recently evolved bacterial pathogens need to be reconsidered [19] and should be based on species-specific short-term molecular clock rates.

Age estimates for viruses depend on the use of archival samples that were stored over several years or decades. Only very few attempts, summarized in Table 2, have been made to estimate ages in bacteria with this approach, in part because their clock rates were thought to be too slow. In the case of *Yersinia pestis* which was introduced to Madagascar in the early 20^th^ century, the clock rate was similar to that of Ochman and Wilson (Table 2). However, a clock rate dated by migration of *Buchnera*, an aphid endosymbiote, to North America in the late 19^th^ century is two orders of magnitude higher (Table 2).

**Table 2:** Published ages and clock rates for microevolution in selected bacteria.

Taxon	Clock rate	Age (yrs)	Citation	Sampling period (yrs)
*Campylobacter jejuni*	μ_S_ = 2.8×10^−5^	400	[20]	3
pandemic *Vibrio cholerae*	μ_S_ = 6.7×10^−5^	130	[21]	34
*Staphylococcus aureus* ST239	μ = 3.3×10^−6^	45	[22]	21
*Yersinia pestis* (Madagascar)	μ = 8.6×10^−9^	100	submitted	70
*H. pylori* serial isolates	μ_S_ = <2×10^−5^	>11,000	[49]	1.8
*Buchnera* (North America)	μ_S_ = 2.2×10^−7^	<135	[71]	extant
*H. pylori* in Pacific	μ = 2.6×10^−7^	70,000	[29]	extant

NOTE: *H. pylori* in Pacific was calculated from the raw output of the ClonalFrame analyses in citation [29] as μ =  theta/2/coalescent unit/concatenated sequence length where theta, the mutation rate x 2, was 720.8 (95% confidence interval [CI] 508.4–985.9), coalescent unit was 400,000 yrs and concatenated sequence length was 3,412 bp. The confidence limits of μ were 1.8×10^−7^ to 3.6×10^−7^.

μ_S_: synonymous clock rate per nucleotide per year.

μ: mutational clock rate per nucleotide per year. The sampling period is designated as extant when date of sampling was not considered in date estimates.

Two recent studies of *Campylobacter jejuni* and *Vibrio cholerae* have found synonymous clock rates of >10^−6^ per site per year, several orders of magnitude higher than the clock rate of Wilson and Ochman. However, we are sceptical about the validity of these two estimates due to problems with their sampling schemes. The *C. jejuni* isolates were obtained over a three year period from infected humans within a sampling area of only 968 km^2^ in Lancashire, England [20], and might reflect admixture due to the import of novel polymorphisms from outside the catchment area. Similarly, the *V. cholerae* estimates were based on a comparison of only three genomes whose epidemiological patterns suggested that they had evolved soon before the dates of sampling [21]. A third recent study found a clock rate of 3×10^−6^ for ST239 of *Staphylococcus aureus*, which would mean that ST239 evolved in the mid-1960's [22]. However, the ST239 genealogy consists of multiple, early radiations, which suggests adaptation due to selective pressures.

Clock rates are distorted when based on polymorphisms that are under positive selection because adaptation can increase the fixation rate for mutations by orders of magnitude [2]. As an extreme example, serial isolates from human infections that are repeatedly treated with antibiotics acquire mutations that are associated with antibiotic resistance and can result in hyper-mutation [23]. 68 mutations in the 6.5 Mbp genome were observed over eight years of lung infection by *Pseudomonas aeruginosa* in a patient with cystic fibrosis [24] and 35 mutations in the 2.9 Mbp genome during 12 weeks of endocarditis caused by *S. aureus* [25]. Similarly, patho-adaptive, transient mutations in an *E. coli* adhesin are selected during infection of the urinary tract but rapidly disappear due to source-sink dynamics [26]. Short-term positive selection may be common because an appreciable fraction of *E. coli* genes show traces of such selection [27].

These various analyses show that mutation rates may be sufficiently high in some bacteria that microevolution can be observed within serial bacterial isolates from individual humans. Here we analyze such microevolution within *Helicobacter pylori. H. pylori* is commonly acquired in childhood, after which, in the absence of antibiotic therapy, it can continue to infect the stomachs of humans over their entire lifespan [28]. *H. pylori* has infected humans for at least 60,000 years because it accompanied anatomically modern humans out of Africa [29]–[33]. *H. pylori* also exhibits an atypically high genetic diversity: every third nucleotide in housekeeping gene fragments is polymorphic in global analyses [30], [34], and the pair-wise synonymous diversity of individual genes ranges from 0.1–0.3 [34]. High genetic diversity can reflect a long evolutionary history but can also result from a high mutation rate. Indeed, the frequency of mutants per cell among natural isolates is approximately 10–100 fold higher in laboratory experiments than for *E. coli* [35], [36], with some variation between individual isolates. That high mutation frequency may reflect the lack of genes encoding the MutHLS1 mismatch repair system [28], [37]. A high mutation rate in the laboratory suggests that the mutational clock rate may also be high during natural infection, possibly facilitating the adaptation of these bacteria to individual human hosts [38]. However, as for most bacteria, robust estimates of the microevolutionary mutation rate are lacking.

In addition to a high mutation rate, recombination is also particularly frequent in *H. pylori*. This conclusion was originally reached on the basis of homoplasy analysis [39]. Although this methodology has been recently criticized [40], recombination is clearly frequent in nature because mosaic imports have been observed, a direct signal for homologous recombination. In laboratory experiments, DNA transformation followed by homologous recombination introduces mosaic stretches of 1.3–3.9 Kbp into the recipient, occasionally interrupted by interspersed segments of recipient DNA sequences that have not been replaced [41], [42]. In nature, mixed infection of individual humans with multiple distinct strains [43]–[48] occurs sufficiently frequently that unambiguous mosaics were detected in serial isolates [49] or isolates from members of a family [47]. Recombination is also indicated by analyses using Structure [33] and the three gamete test [29] on random isolates from diverse global sources. In the analyses of serial isolates [49], the sequences of 10 gene fragments were compared between pairs of strains that were isolated from 26 individuals in Louisiana and Colombia at intervals of 3–36 months (mean 1.8 years). No sequence differences were found in 14 pairs, three pairs of isolates differed by a single nucleotide, and six pairs of isolates differed by eight mosaic stretches. (Four other pairs were excluded from analysis because they either reflected mixed infections with genetically unrelated strains or an infection with a cloud of related isolates whose genetic diversity had arisen prior to infection.) For the 6 pairs of isolates with mosaic stretches, homologous recombination had introduced imports of an average size of 417 bp (CI [95% confidence interval] 259–732) at a rate per nucleotide per year of 6.9×10^−5^ (CI 3.5×10^−5^ to 1.2×10^−4^). The three pairs that differed by a single polymorphism were used to calculate a maximal mutation rate per nucleotide per year of 4.1×10^−5^, but these polymorphisms could not be definitively ascribed to mutation because they might have represented atypically short imports [49].

Here we have reanalyzed the same pairs of isolates plus others that spanned longer time periods. We examined the sequence diversity in 78 gene fragments in order to provide robust short-term clock rates for mutation and recombination. These clock rates were compared to long-term clock rates that were calibrated by the dates of human migrations.

## Results

### Novel nucleotide sequences

We sequenced 78 gene fragments from 97 isolates (Table 3, Table S1, Table S2). Two of these fragments are parts of genes that encode outer membrane proteins and all others are within housekeeping genes. We first sequenced an average of 398 bp from each gene fragment; for fragments with polymorphisms we also sequenced ∼500 bp from each of the flanking regions. This resulted in an average total of 39,301 bp that was sequenced per isolate, almost ten times more than in our previous study [49]. The 97 isolates included 34 pairs of serial isolates from continuously infected individuals, of which 22 had been the subject of our previous analysis [49]. Twelve other pairs were from chronically infected patients in the Netherlands [50] with an average sampling interval of 8.4 years (Table 3). The remaining 29 isolates were from 10 families consisting of siblings plus their parents with an average age of 44.5 years from Colombia (4 families), Korea (3), the UK (2) and the USA (1) [47]. The strains within each pair or group of isolates must have diverged very recently because each pair/group shared identical sequences within at least four of the seven MLST housekeeping fragments. In contrast, in previous population genetic studies based on these seven gene fragments [30], [32], [33], [47], random pairs of isolates were usually distinct at all or most of the seven gene fragments. Despite the limited differences found here between pairs of isolates, the frequency of polymorphic sites across the entire data set was high (0.18±0.04), almost as high as in a comparison of the same 78 gene fragments from seven genomic sequences (0.27±0.07; Table S4).

**Table 3:** Sequence comparisons and sources of isolates.

Category	Details
Number of gene fragments	78
Standard sequence (range)	398 bp (294–627)
Extended sequence (range)	1417 bp (954–1744)
Mean total bp per isolate (range)	39,301 (30,775–60,447)
44 serial isolates: Pairs: mean interval (range)	1–22: 1.8 yrs (0.25–4.0)
24 serial isolates: Pairs: mean interval (range)	33–44: 8.4 yrs (7.4–10.2)
29 family isolates: Groups: mean age (range)	23–32: 44.5 yrs (10–78)

### Sequence comparisons

**Figure 2:**
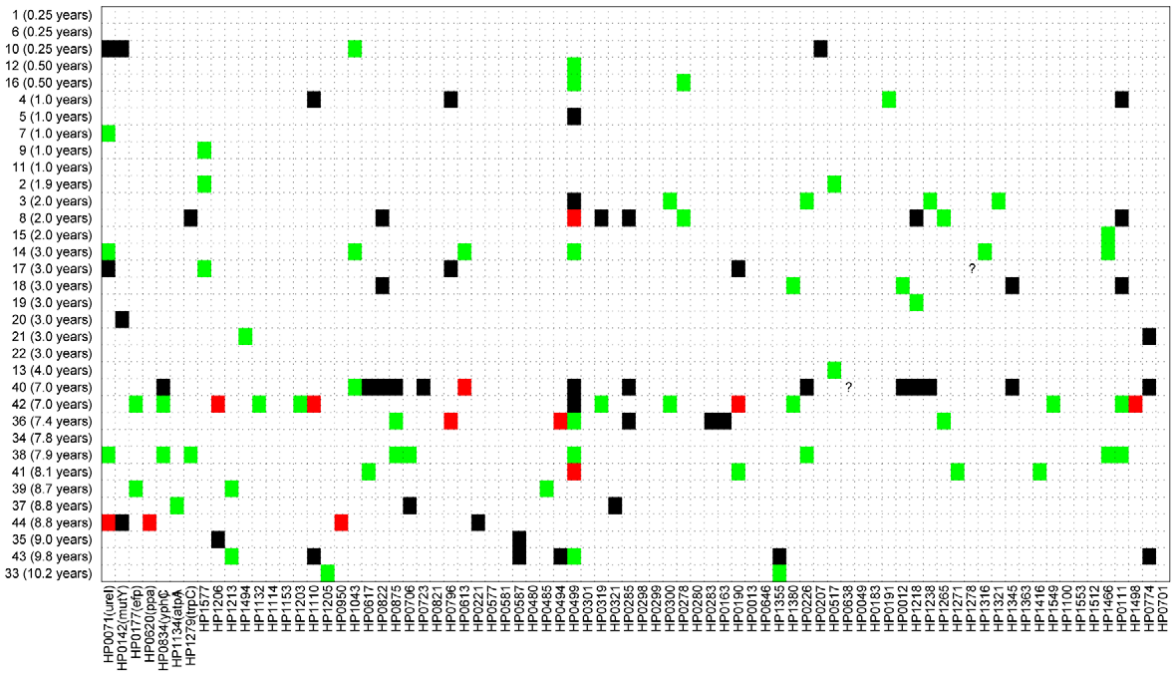
Sequence differences for 78 gene fragments (X axis) that were tested from 34 pairs of sequential isolates (Y axis). Of 2,650 pairs of sequenced gene fragments, 2,526 were identical (white), 62 differ by one polymorphism (green), 12 had two polymorphisms (red), and 50 had at least three (black). Two question marks indicate missing data that were not used for comparisons. Gene fragments are designated by their designations in the genome of 26695 (HPxxxx) [63], except that the first seven gene fragments that are used for MLST of *H. pylori* [34] also include the gene designations.

**Figure 3:**
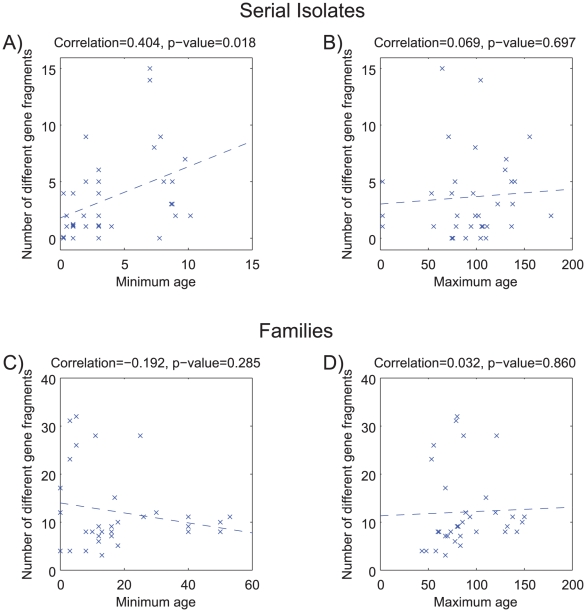
Age versus number of different gene fragments in pairwise comparisons. (A,B) serial isolates. (C, D) isolates within each family. (A) Minimum age was the time separation between pairs of isolates. (B) Maximum age was the sum of the ages of the infected person upon isolation of the serial isolates. (C) Minimum age was the minimum age of the two subjects—20. (D) Maximum age was the sum of the ages of the two family members. Each plot contains a linear regression of the data, whose correlation (R) and probability (*p*) are indicated above the plot.

Figure 2 shows a comparison of the paired sequences from the serial isolates. Out of a total of 2650 pair-wise sequence comparisons of gene fragments, 62 contained 1 polymorphic site, 12 showed two polymorphisms and 50 showed more than two polymorphisms. The total number of fragments with sequence differences correlates significantly with the time difference between the serial samples (*R* = 0.4, *p* = 0.02; Figure 3A), referred to as the minimal age below. Thus, sequence diversity introduced by mutation plus recombination seems to accumulate in a clock-like manner in infected individuals. We note that minimal age represents only a lower bound for the time of divergence between those isolates because the variant might have arisen earlier and persisted together with the parent in the form of a mixed infection. The maximal age is the extreme opposite scenario to the minimal age, namely that the variants evolved soon after birth. We approximated the maximal time of divergence within each individual as the sum of the ages at sampling. There is apparently no correlation between this maximal age and the number of polymorphic fragments (*R* = 0.07, *p* = 0.7, Figure 3B).

**Figure 4:**
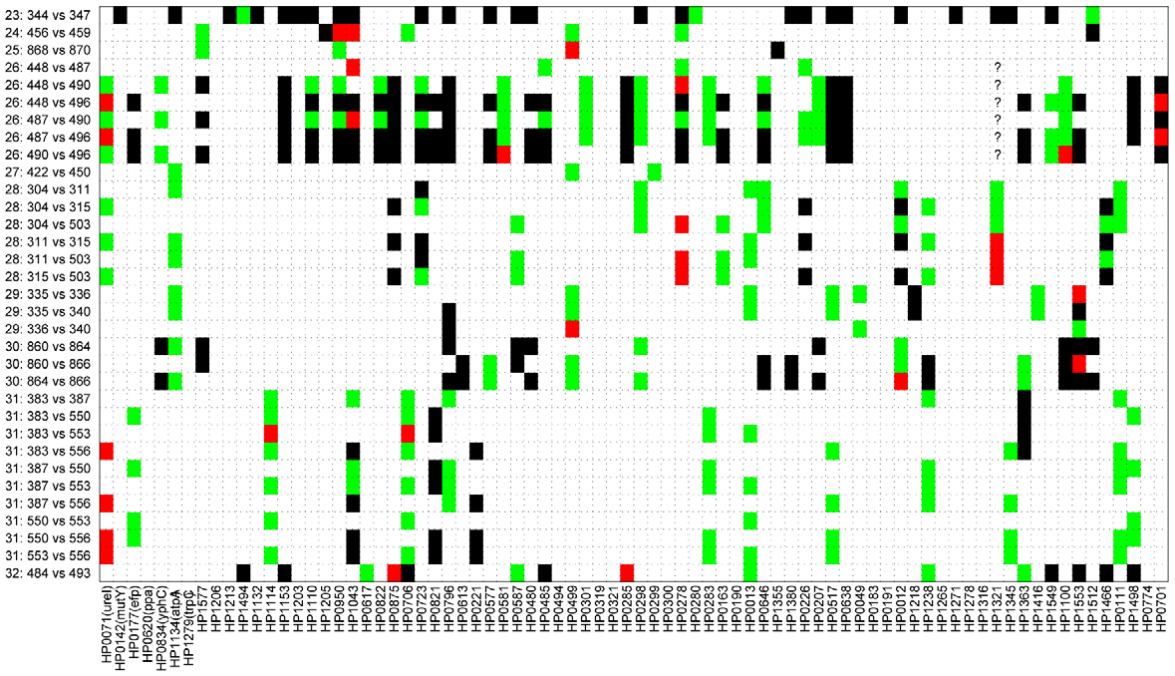
Pair-wise comparison of sequences from 29 isolates acquired from members of 10 families. Of 2,568 sequenced gene fragments, 2,169 were identical (white), 183 had one difference (green), 30 had two differences (red), and 186 had at least four differences (black). Six question marks indicate missing data that were not used for comparisons.

Pair-wise comparisons of sequences from the family isolates revealed even greater diversity (Figure 4), as expected because the time of separation of these pairs is greater. Out of 2568 pair-wise gene fragment comparisons, 183 showed one nucleotide difference, 30 had two and 186 had at least three. However, although the longer time span for divergence of the family isolates was expected to show even stronger correlations with time, this was not the case. Instead, we could not find a significant correlation between the numbers of non-identical gene fragments and any function of the age of the family members that was tested. For example, if infection were transmitted to siblings or children when they reached 20 years of age, a significant correlation should have been observed between the numbers of distinct gene fragment sequences and the minimum age of the two family members – 20 (minimal age), but this was not the case (*R* = −0.19; *p* = 0.28) (Figure 3C). Similarly, if each of the family members were infected at birth, a significant correlation would have been expected against the sum of the ages of the two family members (maximal age), but again this was not the case (*R* = 0.03; *p* = 0.86; Figure 3D). Visual examination of the data indicated that this lack of correlation with age largely reflected two families, numbers 23 and 26, which had unusually high levels of polymorphism. After removal of data from these two families, the number of differences was significantly correlated with maximal age (*R* = 0.4; *p* = 0.045; Figure S1D).

### Model-based analysis

We designed a statistical model of the microevolutionary process in order to analyze our data. Our model assumes that each sequenced fragment evolved independently for an unknown number of years. During that time, mutation events happen according to a molecular clock with a constant rate *m* per site and per year, and independent recombination events occur in and around the fragment at a constant rate *r* per initiation site and per year. We follow Falush *et al.* [49] in assuming that when a recombination event happens, it affects a stretch of DNA with a geometrically distributed length of mean λ from the initiation point. In the affected region, each site has a probability of being substituted which is drawn from a normal distribution with mean equal to ν. Our recombination model is therefore similar to that of ClonalFrame [51], except that the rate of substitution introduced by each recombination event is drawn from a distribution rather than being constant. The use of such a distribution is advantageous because it reflects the diversity of the level of relatedness between donor and recipient for all recombination events.

We applied this microevolutionary model to our data using Approximate Bayesian Computation (ABC). ABC is a Monte-Carlo method to perform statistical inference on the parameters of a model using summary statistics [52], and is well suited to deal with the complex models that arise in population genetics [53]–[55]. We therefore performed ABC inference under the model described above, using the algorithm described by Marjoram *et al.* [56]. This algorithm uses a Monte-Carlo Markov Chain, but instead of guiding the random walk on the parameter space according to the likelihood, as is usually done, it is guided according to the ability of the parameters to produce a dataset with similar summary statistics (see Materials and Methods).

Our model can be directly applied to the serial isolate data since it describes the evolution between a pair of isolates, resulting in the parameter estimates that are summarized in the first column of Table 4. However, we also wanted to perform the same statistical analysis with the family isolate data as for the serial isolate data. To do so, we first attempted to deduce the genealogical relationships between the isolates within each family using ClonalFrame [51], but the statistical uncertainty found in these reconstructions was too high to make this approach practical, i.e. it is unclear who infected whom. Therefore, we made no assumptions about phylogeny but rather performed pair-wise comparisons of each pair of isolates within a family. This technique has the disadvantage that it might count some microevolutionary events several times in the pair-wise comparisons, but it is the only approach available in the absence of a robust estimate of phylogenies. The parameter estimates for the family data are also reported in Table 4.

**Table 4:** Estimated average [95% credibility intervals] of parameters from ABC analysis.

Parameter	Serial Isolates	Family Isolates
*m* (mutation rate)	1.36×10^−6^ [0.88×10^−6^;1.89×10^−6^]	4.51×10^−6^ [3.48×10^−6^;5.40×10^−6^]
*r* (recombination rate)	2.44×10^−7^ [1.74×10^−7^;3.45×10^−7^]	8.07×10^−7^ [5.95×10^−7^;10.42×10^−7^]
λ (tract length, bp)	1,247 [841;1721]	1,419 [1008;1763]
ν (polymorphism rate)	0.016 [0.006;0.026]	0.022 [0.017;0.028]
ν ⋅ λ (polymorphisms)	18.62 [7.67;28.03]	30.96 [20.91;46.13]
*r*/*m*	0.19 [0.11;0.26]	0.18 [0.13;0.25]
*r* ⋅ *λ* ⋅ *ν*/*m*	3.35 [1.66;5.56]	5.49 [3.46;8.06]

### Model validation

**Figure 5:**
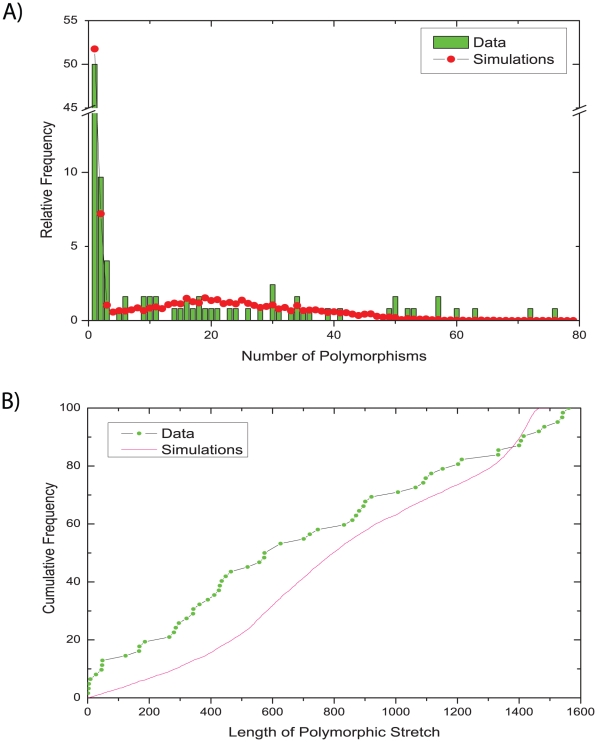
Comparisons of observed and simulated data for sequences from serial isolates. (A) relative frequency of numbers of polymorphisms. (B) cumulative frequency of the lengths of polymorphic stretches. Comparable data for the family isolates are presented in Figure S2.

We assessed the validity of our model by comparing the observed distributions for two summary statistics that were not used in the ABC inference with their posterior predictive distributions [57], i.e. the distribution obtained by simulations using parameters from the posterior sample (Figure 5). This method of model criticism has been applied previously in multiple ABC studies [58], [59]. The distribution of the number of polymorphisms per gene fragment was quite similar between the data and the posterior simulations from the serial isolates: most gene fragments contained only one polymorphism, several contained two or three polymorphisms, and the frequencies of larger numbers of polymorphisms were spread fairly uniformly over the entire data set (Figure 5A). The length of the polymorphic stretches was less uniform (Figure 5B). The data contained multiple fragments with polymorphisms in stretches of less than 50 bp whereas larger polymorphic stretches were distributed fairly evenly up to the maximum length of just under 1,600 bp. In contrast, the posterior predictive distribution of lengths of polymorphic stretches was fairly uniform, except that stretches of 500–900 bp and of 1,300–1,500 bp were somewhat more frequent. However, these differences between observed data and simulations were relatively minor, again providing support for the validity of our model and inference methodology. Similarly, only minor differences were found when comparing the family data in the same way (Figure S2).

## Discussion

### Parameter estimates for the sequential isolate data

The average rate of polymorphism introduced by recombination events (ν) was 0.02 (Table 4), which is somewhat lower than the average genetic distance between unrelated members of *H. pylori* from Ladakh in northern India (0.03) [32] or Europe (0.04) [33]. In turn, this lower rate indicates that donors and recipients were somewhat more closely related than are random, unrelated isolates, and may reflect increased opportunities for recombination within members of the same subpopulations due to geographical structure [47]. Local geographic structure arises due to isolation by distance [30] and isolates within families may have had more opportunities for prior recombination events that would reduce diversity than do geographically separated isolates.

The mean length of imports (λ) was 1247 bp, which is in good agreement with recent estimates from experimental work [41], [42], but considerably greater than the value of 417 bp found previously among serial isolates by Falush *et al.* [49]. We ascribe this discrepancy to the limited number (eight) of recombination events examined by Falush *et al.* rather than to differences in methodology. The combination of these two estimates (λ ⋅ ν) indicates that on average 18.6 nucleotide substitutions were introduced by each recombination event, although this number ranged greatly between individual recombination events (Figure 5A).

The average rate of mutation *m* (per nucleotide site, per year) was estimated as 1.4×10^−6^ and the average rate of recombination *r* (per initiation site, per year) was 2.4×10^−7^. These estimates are sensitive to our choice of prior for the evolutionary time of split between isolates, on which there is much uncertainty. However, Figure 3A provides support for clock-like microevolution versus the time of isolation of the paired isolates (minimal age) and the ABC analyses were performed using very uninformative priors for their time of separation, consisting of the range since birth to the time of isolation of the bacterial strains. Furthermore, data and simulations based on the estimated parameters correspond well in regard to the frequencies of numbers of polymorphisms and reasonably well for the lengths of polymorphic stretches (Figure 5). We therefore conclude that these estimates are reasonably accurate as measures of mutation and recombination rates over very short time periods of up to 10 years.

The ratio *r*/*m* should be a robust measure of the relative frequencies at which mutation and recombination are initiated at a given site because both *r* and *m* are equally affected by any under- or over-estimation of the split times. The mean estimate for *r*/*m* is 0.19. Thus mutations are on average 5 times more frequent than recombination events over the genome of *H. pylori*. However, even though it happens less often than does mutation, the effect of recombination is much more dramatic than that of mutation, as indicated in Table 4 by the estimate of 3.4 for *r* ⋅ λ ⋅ ν/*m*, which represents the ratio of rates at which a site is substituted through recombination and mutation. According to this estimate, a site is >3 times as likely to be substituted by recombination than by mutation.

### Parameter estimates for the family isolate data

The average estimates for *m* and *r* were about 3 times higher within the families than in the paired isolates (Table 4). We considered the possibility that the different estimates of *r* and *m* between serial and family data might reflect the fact that families 23 and 26 exhibited elevated numbers of polymorphisms. However, after excluding these two families, the resulting parameter estimates did not differ dramatically from the estimates summarized in Table 4. We note, however, that in the absence of specific evidence from the data, Bayesian analysis with a broad uniform prior will tend to settle on values within the range of the prior rather than at the extremes. Genetic diversity within families correlated with maximal age (after excluding families 23 and 26; Figure S1D) whereas diversity between serial isolates correlated with minimal age (Figure 3A). Thus, this tendency to use internal values within a broad prior range would shift our parameter estimates for the serial and family isolates in opposite directions away from the extreme age that best correlated with diversity, and could well account for the threefold difference between the two sets of parameter estimates. Finally, we also note that we tested 10 family isolates to see whether the elevated numbers of polymorphisms in families 23 and 26 were accompanied by extreme *in vitro* frequencies of mutation and DNA transformation (from strain J99). However, although a broad range was measured for the frequencies of both mutation (sevenfold) and transformation (200 fold) (each with one outlier), there was no clear correlation between the two exceptional families and the extremes of the laboratory rates (data not shown).

In contrast to *r* and *m* themselves, the ratio *r*/*m* is independent of time and should be robust. This ratio has a mean value of 0.18, very similar to the estimate of 0.19 for the serial isolates (Table 4). Similarly, the tract length λ and the frequency ν at which polymorphisms were introduced are also independent of time, and were only slightly higher in the family data than in the serial isolate data (Table 4). ν remains lower than the average pair-wise distance between two random strains of *H. pylori* and λ is consistent with recent estimates of tract lengths introduced by recombination in the laboratory [41], [42]. Finally, the relative effect of recombination and mutation, *r* ⋅ *λ* ⋅ *ν*/*m*, should also be relatively robust in regard to uncertainties about time of separation. The mean value of 5.5 was 50% higher than for the serial isolates (3.4), possibly reflecting more opportunities for recombination over the longer time period of infection in the families than within the serial isolates.

### Mutation rates in *H. pylori* versus other bacteria

The estimated short-term mutation rates in the serial and family isolates were 1.4×10^−6^ and 4.5×10^−6^, respectively. This range is a robust estimate of the mutation rate over years to decades. It is also possible to calculate a longer term mutation rate for genetic diversity between *H. pylori* from different global sources, because isolation by distance over the last 60,000 years has resulted in parallel trends in changes in genetic diversity between these bacteria and their human hosts [30]. As a result, diversity between *H. pylori* from different global sources has accumulated in a clock-like manner that correlates with, and can be dated by, the times of separation of their human hosts [29]. We estimated the long-term mutation rate on the basis of the ClonalFrame analyses described by Moodley *et al*. [29], yielding a long-term estimate for *m* of 2.6×10^−7^ (Table 2). This value is 5–17 fold lower than the short-term rates calculated here, which is probably a general phenomenon among bacteria according to theoretical considerations [14], [15]. One reason for such discrepancies is that even neutral polymorphisms are usually lost over time through genetic drift. A second reason is that non-synonymous mutations will be selected against with time because many of them are slightly deleterious, which should result in a lower *d_N_/d_S_* ratio, the relative rates of non-synonymous to synonymous mutations. A loss of non-synonymous mutations will reduce the apparent mutation rate because approximately 75% of all mutations in coding genes are non-synonymous.

We estimated what proportion of the 5–17 fold reduction in the long-term mutation rate could be accounted for by the loss of non-synonymous mutations. Based on our simulations with the serial isolates, approximately 99% of paired fragments with only one polymorphism resulted from mutation rather than recombination. Thus we could equate the polymorphisms within fragments containing only one SNP to mutations, allowing the calculation of *d_N_/d_S_* even when other fragments had undergone recombination. The resulting *d_N_/d_S_* ratio was 0.5, which indicates that only little purifying selection had taken place over the time period considered here, as is also the case in other examples of recent microevolution [2], [22]. Over longer time periods, purifying selection of deleterious non-synonymous mutations does take place in *H. pylori*, resulting in an average *d_N_/d_S_* ratio of 0.07 (sevenfold lower) in housekeeping genes among unrelated isolates [34], which is in good agreement with the 5–17 fold difference in mutation rates.

Finally, we return to the general question of the short-term clock rate within bacteria. The results presented here demonstrate that the short-term clock rate in *H. pylori* is approximately the same (0.4–1.4 fold) as the short-term clock rate in *S. aureus* ST239, 6.2–20.5 times the rate in *Buchnera* and 158–524 times the rate in *Y. pestis* (Table 2). These comparisons show that the short-term clock rate varies dramatically among different bacteria, and in some cases overlaps with those of RNA viruses [60]. However, in all cases considered here, it is higher than the long-term (synonymous) clock rate of 3.4×10^−9^ that has often been used until now to calculate the ages of genetically monomorphic bacteria.

## Materials and Methods

### Bacterial isolates

We studied two types of bacterial isolates of *H. pylori*: serial isolates which were collected from individual persons after a specified time interval, and family isolates which were collected concurrently from two or more members of the same family (Table S2). The 68 serial isolates were collected from 34 patients at intervals ranging from 3 months to 10.2 years. The 29 family isolates were collected from 2 to 5 members of 10 families.

### Nucleotide sequencing

Fragments of 78 genes were sequenced (Table S1). Additional extended flanking regions were also sequenced when sequence polymorphisms were detected in the standard fragments. PCR products were amplified and sequences were performed by standard Sanger sequencing on an ABI 3730 XL as described [47] using the oligonucleotide primers listed in Table S3, except that PCR products were cleaned by using shrimp alkaline phosphatase plus exonuclease I. All sequence data has been deposited in the *Helicobacter pylori* Multi Locus Sequence Typing website (http://pubmlst.org/helicobacter/projects/microevolution/alldata.zip) developed by Keith Jolley and sited at the University of Oxford [61].

### Microevolutionary model

We designed a microevolutionary model which describes the evolution of the genome of a strain over a certain period of time *T*. During this time, each nucleotide of the genome is mutated with probability *T*×*m* and is the initiation site of a recombination with probability *T*×*r*. When a recombination occurs, it affects a segment of the genome starting from the initiation site and stretching to the right over a length which is geometrically distributed with mean λ. Each site of the affected segment has a probability to be substituted which is normally distributed with mean ν.

The parameters of this microevolutionary model are the time *T* separating each compared pair of isolates, the mutation rate *m* per site per year, the recombination rate *r* per initiation site per year, the average tract length of recombination λ and the average rate of polymorphism introduced by recombination ν. The prior for the time of divergence between the paired isolates is described below. Priors for the four other parameters were uniform from 0 to infinity (improper prior).

### Prior on the evolutionary time separating pairs of isolates

Because the evolutionary time separating pairs of isolates is unknown, we had to assume a prior for this quantity in order to perform Bayesian inference. For the serial isolates, we know that the time spent between successive isolations represents a lower bound. If we further assume that the two isolates originated from the same infection, and since this infection must have happened after the birth of the patient, we get an upper bound equal to twice the age of the infected person. We thus assumed a uniform prior for the evolutionary time separating serial isolates between these lower and upper bounds.

For the evolutionary time separating a pair of family isolates, we took a lower bound equal to the minimum of the ages of the two family members minus 20, based on the idea that *H. pylori* infection usually occurs before the age of 20. We took an upper bound equal to the sum of the ages of the two family members. We assumed a uniform prior for the evolutionary time separating pairs of family isolates between these lower and upper bounds.

### Approximate Bayesian Computation analysis

We performed inference under the model above using the Approximate Bayesian Computation (ABC) algorithm described by Marjoram *et al.* [56]. This algorithm was run independently for the serial isolates and the family isolates. The length of each run was set at 100,000 iterations, which took approximately 5 hours on a Desktop computer. Several independent runs were performed and compared manually in order to ensure that good convergence and mixing properties were achieved.

One essential step in ABC analysis is the choice of the summary statistics used, which determines how exact the inference is [52]. If the whole data were used as a summary, the algorithm would be exact but unfeasibly slow. If no summary statistic were used at all, the Markov chain would explore the prior on the parameters. It is thus important to find a handful of statistics that summarize the information contained in the data about the parameters as well as possible. Here we found that the data was well summarized by the numbers of gene fragments with zero, one, two or at least three substitutions, and the average spread of substitutions for the fragments with at least 3 substitutions. The rationale behind this choice is that fragments with one substitution are likely to be caused by mutation whereas fragments with at least 3 substitutions are likely to be caused by recombination. Therefore, even though our model makes no assumption about the cause of observed polymorphisms, the number of fragments with one substitution is informative about the mutation rate *m* and the number of fragments with at least 3 substitutions is informative about the recombination rate *r*. Furthermore, the average spread of substitutions for the fragments with at least 3 substitutions is informative about the average tract length of recombination λ.

We note that this model determines mutation and recombination by a phylogenetic approach, which implicitly assumes that each mutation is fixed rather than resulting in a polymorphism. This approach allows comparisons with the other mutation rates in Table 2, which were also calculated by a phylogenetic approach, except *C. jejuni*. However, as pointed out by one of the reviewers, Joshua B. Plotkin, the sequence differences we have analyzed correspond to segregating polymorphisms, which might have implications for our estimated mutation rates [16], [17], [62].

## Supporting Information

Figure S1 As in Figure 3, except that pair-wise comparisons between isolates from families 23 and 26 were not included in (C,D).
(0.16 MB PDF)

Figure S2 Comparisons of data and simulations from family isolates. All other details are as in Figure 5.
(0.27 MB PDF)

Table S1 78 gene fragments whose sequences were compared between paired isolates and within isolates from families.
(0.04 MB XLS)

Table S2 (A) Paired serial isolates from 34 individuals. (B) Single isolates from 29 individuals in 10 families.
(0.03 MB XLS)

Table S3 Sequences of oligonucleotide primers used for amplification and sequencing.
(0.09 MB XLS)

Table S4 Polymorphic sites in 78 gene fragments from genomic sequences and from the paired isolates.
(0.06 MB XLS)

## References

[1] Achtman M, Morelli G, Zhu P, Wirth T, Diehl I (2004). Microevolution and history of the plague bacillus, *Yersinia pestis*.. Proc Natl Acad Sci (USA).

[2] Roumagnac P, Weill F-X, Dolecek C, Baker S, Brisse S (2006). Evolutionary history of *Salmonella* Typhi.. Science.

[3] Nübel U, Roumagnac P, Feldkamp M, Song JH, Ko KS (2008). Frequent emergence and limited geographic dispersal of methicillin-resistant *Staphylococcus aureus*.. Proc Natl Acad Sci (USA).

[4] Sreevatsan S, Pan X, Stockbauer K, Connell ND, Kreiswirth BN (1997). Restricted structural gene polymorphism in the *Mycobacterium tuberculosis* complex indicates evolutionarily recent global dissemination.. Proc Natl Acad Sci (U S A).

[5] Pupo GM, Lan R, Reeves PR (2000). Multiple independent origins of Shigella clones of *Escherichia coli* and convergent evolution of many of their characteristics.. Proc Natl Acad Sci (U S A).

[6] Leopold SR, Magrini V, Holt NJ, Shaikh N, Mardis ER (2009). A precise reconstruction of the emergence and constrained radiations of *Escherichia coli* O157 portrayed by backbone concatenomic analysis.. Proc Natl Acad Sci (USA).

[7] Parkhill J, Sebaihia M, Preston A, Murphy LD, Thomson N (2003). Comparative analysis of the genome sequences of *Bordetella pertussis*, *Bordetella parapertussis* and *Bordetella bronchiseptica*.. Nature Genet.

[8] Wirth T, Morelli G, Kusecek B, Van Belkum A, van der Schee C (2007). The rise and spread of a new pathogen: seroresistant *Moraxella catarrhalis*.. Genome Research.

[9] Holmes EC (2008). Evolutionary history and phylogeography of human viruses.. Annu Rev Microbiol.

[10] Ochman H, Wilson AC (1987). Evolution in bacteria: Evidence for a universal substitution rate in cellular genomes.. J Mol Evol.

[11] Sheridan PP, Freeman KH, Brenchley JE (2003). Estimated minimal divergence times of the major bacterial and archaeal phyla.. Geomicrobiology Journal.

[12] Wirth T, Falush D, Lan R, Colles F, Mensa P (2006). Sex and virulence in *Escherichia coli*: an evolutionary perspective.. Mol Microbiol.

[13] Battistuzzi FU, Feijao A, Hedges SB (2004). A genomic timescale of prokaryote evolution: insights into the origin of methanogenesis, phototrophy, and the colonization of land.. BMC Evol Biol.

[14] Ho SY, Larson G (2006). Molecular clocks: when times are a-changin'.. Trends Genet.

[15] Ho SY, Shapiro B, Phillips MJ, Cooper A, Drummond AJ (2007). Evidence for time dependency of molecular rate estimates.. Syst Biol.

[16] Rocha EP, Smith JM, Hurst LD, Holden MT, Cooper JE (2006). Comparisons of dN/dS are time dependent for closely related bacterial genomes.. J theor Biol.

[17] Kryazhimskiy S, Plotkin JB (2008). The population genetics of dN/dS.. PLoS Genet.

[18] Ochman H, Elwyn S, Moran NA (1999). Calibrating bacterial evolution.. Proc Natl Acad Sci (U S A).

[19] Achtman M (2008). Evolution, population structure and phylogeography of genetically monomorphic bacterial pathogens.. Annu Rev Microbiol.

[20] Wilson DJ, Gabriel E, Leatherbarrow AJ, Cheesbrough J, Gee S (2009). Rapid evolution and the importance of recombination to the gastroenteric pathogen *Campylobacter jejuni*.. Mol Biol Evol.

[21] Feng L, Reeves PR, Lan R, Ren Y, Gao C (2008). A recalibrated molecular clock and independent origins for the cholera pandemic clones.. PLoS ONE.

[22] Harris SR, Feil EJ, Holden MT, Quail MA, Nickerson EK (2010). Evolution of MRSA during hospital transmission and intercontinental spread.. Science.

[23] Mena A, Smith EE, Burns JL, Speert DP, Moskowitz SM (2008). Genetic adaptation of *Pseudomonas aeruginosa* to the airways of cystic fibrosis patients is catalyzed by hypermutation.. J Bacteriol.

[24] Smith EE, Buckley DG, Wu Z, Saenphimmachak C, Hoffman LR (2006). Genetic adaptation by *Pseudomonas aeruginosa* to the airways of cystic fibrosis patients.. Proc Natl Acad Sci (USA).

[25] Mwangi MM, Wu SW, Zhou Y, Sieradzki K, De Lencastre H (2007). Tracking the in vivo evolution of multidrug resistance in *Staphylococcus aureus* by whole-genome sequencing.. Proc Natl Acad Sci (USA).

[26] Weissman SJ, Beskhlebnaya V, Chesnokova V, Chattopadhyay S, Stamm WE (2007). Differential stability and trade-off effects of pathoadaptive mutations in the *Escherichia coli* FimH adhesin.. Infect Immun.

[27] Chattopadhyay S, Weissman SJ, Minin VN, Russo TA, Dykhuizen DE (2009). High frequency of hotspot mutations in core genes of *Escherichia coli* due to short-term positive selection.. Proc Natl Acad Sci (USA).

[28] Suerbaum S, Josenhans C (2007). *Helicobacter pylori* evolution and phenotypic diversification in a changing host.. Nat Rev Microbiol.

[29] Moodley Y, Linz B, Yamaoka Y, Windsor HM, Breurec S (2009). The peopling of the Pacific from a bacterial perspective.. Science.

[30] Linz B, Balloux F, Moodley Y, Manica A, Liu H (2007). An African origin for the intimate association between humans and *Helicobacter pylori*.. Nature.

[31] Eppinger M, Baar C, Linz B, Raddatz G, Lanz C (2006). Who ate whom? Adaptive *Helicobacter* genomic changes that accompanied a host jump from early humans to large felines.. PLoS Genet.

[32] Wirth T, Wang X, Linz B, Novick RP, Lum JK (2004). Distinguishing human ethnic groups by means of sequences from *Helicobacter pylori*: lessons from Ladakh.. Proc Natl Acad Sci (USA).

[33] Falush D, Wirth T, Linz B, Pritchard JK, Stephens M (2003). Traces of human migrations in *Helicobacter pylori* populations.. Science.

[34] Achtman M, Azuma T, Berg DE, Ito Y, Morelli G (1999). Recombination and clonal groupings within *Helicobacter pylori* from different geographical regions.. Mol Microbiol.

[35] Bjorkholm B, Sjolund M, Falk PG, Berg OG, Engstrand L (2001). Mutation frequency and biological cost of antibiotic resistance in *Helicobacter pylori*.. Proc Natl Acad Sci (USA).

[36] Kang JM, Iovine NM, Blaser MJ (2006). A paradigm for direct stress-induced mutation in prokaryotes.. FASEB J.

[37] Lin Z, Nei M, Ma H (2007). The origins and early evolution of DNA mismatch repair genes–multiple horizontal gene transfers and co-evolution.. Nucleic Acids Res.

[38] Kang J, Blaser MJ (2006). Bacterial populations as perfect gases: genomic integrity and diversification tensions in *Helicobacter pylori*.. Nat Rev Microbiol.

[39] Suerbaum S, Maynard Smith J, Bapumia K, Morelli G, Smith NH (1998). Free recombination within *Helicobacter pylori*.. Proc Natl Acad Sci (U S A).

[40] Meinersmann RJ, Romero-Gallo J, Blaser MJ (2008). Rate heterogeneity in the evolution of *Helicobacter pylori* and the behavior of homoplastic sites.. Infect Genet Evol.

[41] Kulick S, Moccia C, Didelot X, Falush D, Kraft C (2008). Mosaic DNA imports with interspersions of recipient sequence after natural transformation of *Helicobacter pylori*.. PLoS ONE.

[42] Lin EA, Zhang XS, Levine SM, Gill SR, Falush D (2009). Natural transformation of *Helicobacter pylori* involves the integration of short DNA fragments interrupted by gaps of variable size.. PLoS Pathog.

[43] Taylor NS, Fox JG, Akopyants NS, Berg DE, Thompson N (1995). Long-term colonization with single and multiple strains of *Helicobacter pylori* assessed by DNA fingerprinting.. J Clin Microbiol.

[44] Berg DE, Gilman RH, Lelwala-Guruge J, Srivastava K, Valdez Y (1997). *Helicobacter pylori* populations in Peruvian patients.. Clin Infect Dis.

[45] Kersulyte D, Chalkauskas H, Berg DE (1999). Emergence of recombinant strains of *Helicobacter pylori* during human infection.. Mol Microbiol.

[46] Raymond J, Thiberg JM, Chevalier C, Kalach N, Bergeret M (2004). Genetic and transmission analysis of *Helicobacter pylori* strains within a family.. Emerg Infect Dis.

[47] Schwarz S, Morelli G, Kusecek B, Manica A, Balloux F (2008). Horizontal versus familial transmission of *Helicobacter pylori*.. PLoS Pathog.

[48] Talarico S, Gold BD, Fero J, Thompson DT, Guarner J (2009). Pediatric *Helicobacter pylori* isolates display distinct gene coding capacities and virulence gene marker profiles.. J Clin Microbiol.

[49] Falush D, Kraft C, Taylor NS, Correa P, Fox JG (2001). Recombination and mutation during long-term gastric colonization by *Helicobacter pylori*: Estimates of clock rates, recombination size and minimal age.. Proc Natl Acad Sci (U S A).

[50] Kuipers EJ, Israel DA, Kusters JG, Gerrits MM, Weel J (2000). Quasispecies development of *Helicobacter pylori* observed in paired isolates obtained years apart from the same host.. J Infect Dis.

[51] Didelot X, Falush D (2007). Inference of bacterial microevolution using multilocus sequence data.. Genetics.

[52] Beaumont MA, Zhang WY, Balding DJ (2002). Approximate Bayesian computation in population genetics.. Genetics.

[53] Pritchard JK, Seielstad MT, Perez-Lezaun A, Feldman MW (1999). Population growth of human Y chromosomes: a study of Y chromosome microsatellites.. Mol Biol Evol.

[54] Tavare S, Marshall CR, Will O, Soligo C, Martin RD (2002). Using the fossil record to estimate the age of the last common ancestor of extant primates.. Nature.

[55] Voight BF, Adams AM, Frisse LA, Qian Y, Hudson RR (2005). Interrogating multiple aspects of variation in a full resequencing data set to infer human population size changes.. Proc Natl Acad Sci (USA).

[56] Marjoram P, Molitor J, Plagnol V, Tavare S (2003). Markov chain Monte Carlo without likelihoods.. Proc Natl Acad Sci (USA).

[57] Gelman A, Meng XL, Stern H (1996). Posterior predictive assessment of model fitness via realized discrepancies.. Statistica Sinica.

[58] Ingvarsson PK (2008). Multilocus patterns of nucleotide polymorphism and the demographic history of *Populus tremula*.. Genetics.

[59] Thornton K, Andolfatto P (2006). Approximate Bayesian inference reveals evidence for a recent, severe bottleneck in a Netherlands population of *Drosophila melanogaster*.. Genetics.

[60] Holmes EC (2010). The comparative genomics of viral emergence.. Proc Natl Acad Sci (USA).

[61] Jolley KA, Chan MS, Maiden MC (2004). mlstdbNet - distributed multi-locus sequence typing (MLST) databases.. BMC Bioinformatics.

[62] Peterson GI, Masel J (2009). Quantitative prediction of molecular clock and K*a*/K*s* at short timescales.. Mol Biol Evol.

[63] Tomb JF, White O, Kerlavage AR, Clayton RA, Sutton GG (1997). The complete genome sequence of the gastric pathogen *Helicobacter pylori*.. Nature.

[64] Tomitani A, Knoll AH, Cavanaugh CM, Ohno T (2006). The evolutionary diversification of cyanobacteria: molecular-phylogenetic and paleontological perspectives.. Proc Natl Acad Sci (USA).

[65] Butterfield NJ (2000). *Bangiomorpha pubescens* n. gen., n. sp.: implications for the evolution of sex, multicellularity, and the Mesoproterozoic/Neoproterozoic radiation of eukaryotes.. Paleobiology.

[66] Holland HD (2002). Volcanic gases, black smokers, and the Great Oxidation Event.. Geochimica et Cosmochimica Acta.

[67] Javaux EJ, Knoll AH, Walter MR (2001). Morphological and ecological complexity in early eukaryotic ecosystems.. Nature.

[68] Date-a-Clade service for the molecular tree of life (2009). http://www.fossilrecord.net/dateaclade/clade_crown_Bilateria.html.

[69] Date-a-Clade service for the molecular tree of life (2009). http://www.fossilrecord.net/dateaclade/clade-platypus-elephant.html.

[70] Tree of Life web project (2009). http://tolweb.org/Fabaceae/21093.

[71] Moran NA, McLaughlin HJ, Sorek R (2009). The dynamics and time scale of ongoing genomic erosion in symbiotic bacteria.. Science.

