# Supplementary material for: Microevolution of Helicobacter pylori during Prolonged Infection of Single Hosts and within Families
Source: PLoS Genet. 2010 Jul 22;6(7):e1001036. doi: 10.1371/journal.pgen.1001036 (PMC2908706; doi:10.1371/journal.pgen.1001036)
Supplement: Figure S1 — As in Figure 3, except that pair-wise comparisons between isolates from families 23 and 26 were not included in (C,D). (0.16 MB PDF) [file pgen.1001036.s001.pdf]

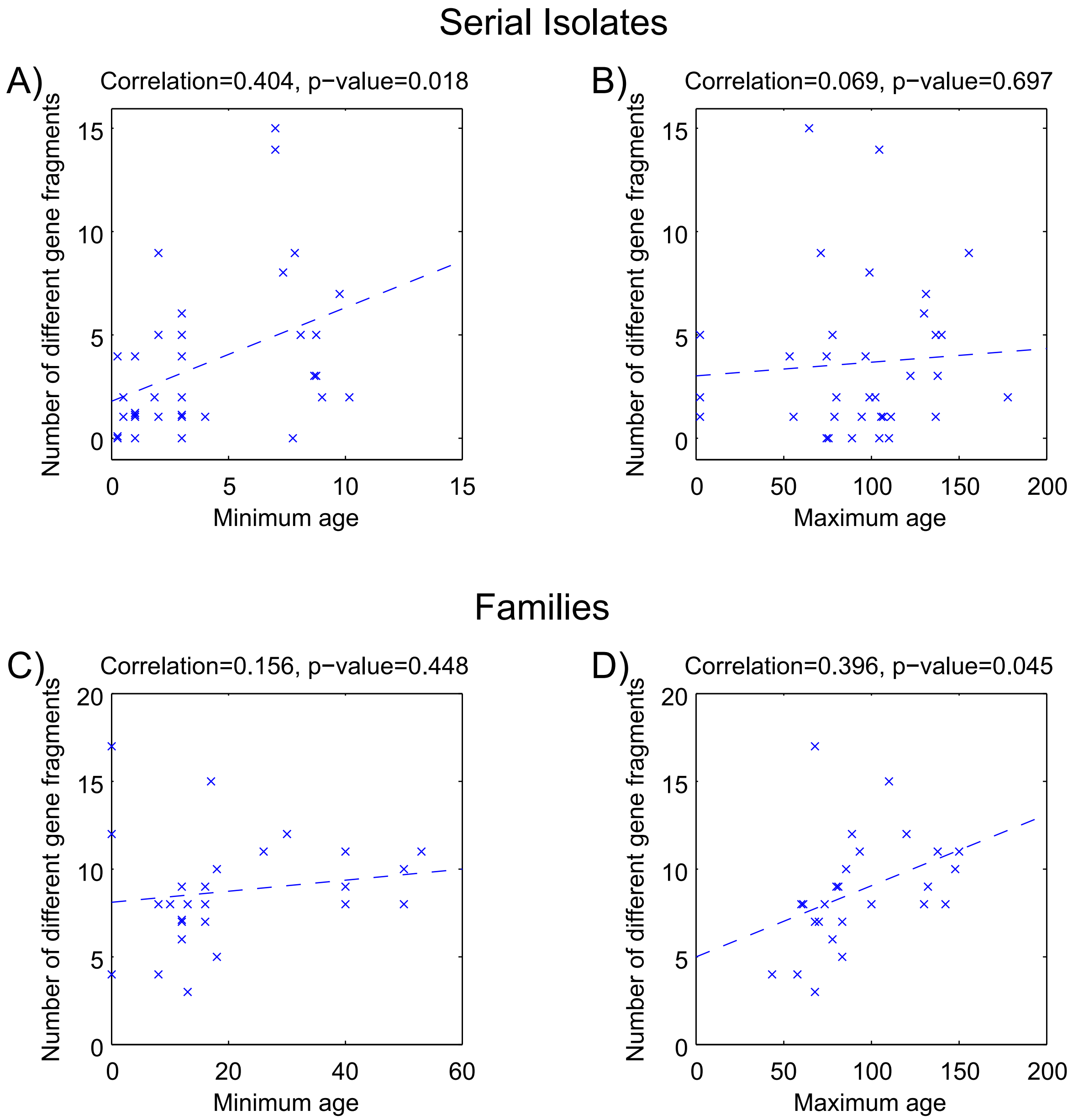

Figure S1. As in Fig. 3, except that pair-wise comparisons between isolates from families 23 and 26 were not included in parts C and D.
