# Supplementary material for: Microevolution of Helicobacter pylori during Prolonged Infection of Single Hosts and within Families
Source: PLoS Genet. 2010 Jul 22;6(7):e1001036. doi: 10.1371/journal.pgen.1001036 (PMC2908706; doi:10.1371/journal.pgen.1001036)
Supplement: Figure S2 — Comparisons of data and simulations from family isolates. All other details are as in Figure 5. (0.27 MB PDF) [file pgen.1001036.s002.pdf]

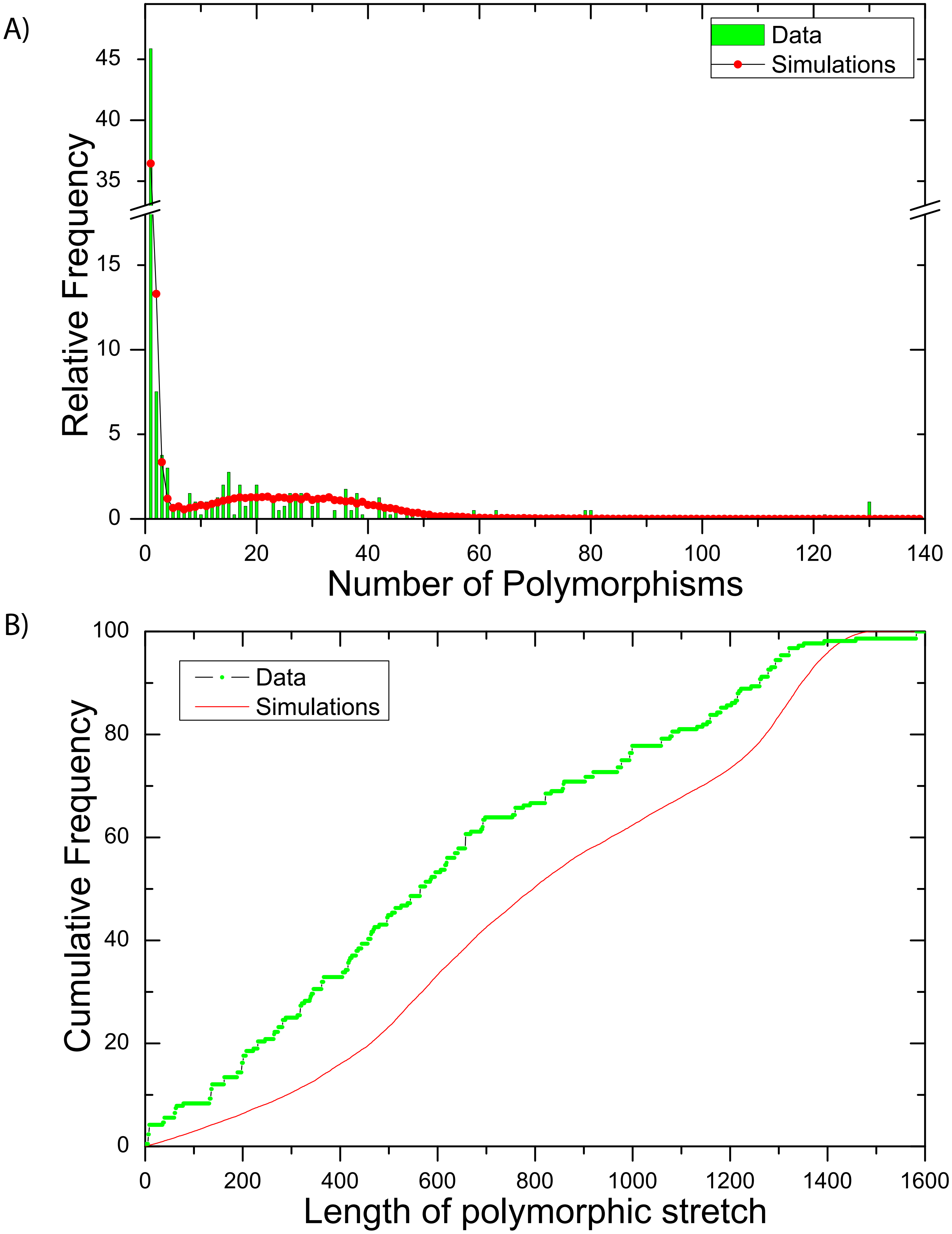

Figure S2. Comparisons of data and simulations from family isolates. All other details are as in Fig. 5.
